# Paracecal hernia due to membranous adhesion of the omentum to the right paracolic gutter

**DOI:** 10.1186/s40792-019-0749-8

**Published:** 2019-11-27

**Authors:** Taro Yokota, Kazuhiro Otani, Junichi Yoshida, Naoki Mochidome, Eiji Miyatake, Chihiro Nakahara, Toshiyuki Ishimitsu, Masao Tanaka

**Affiliations:** Department of Surgery, Shimonoseki City Hospital, 1-13-1 Koyo-cho, Shimonoseki, Yamaguchi 750-8520 Japan

**Keywords:** Paracecal hernia, Internal hernia, Paracolic gutter, Bowel obstruction, Membranous adhesion of the omentum

## Abstract

**Background:**

Paracecal hernias, also known as pericecal hernias, are an exceptionally rare type of internal hernia. We report a unique case of paracecal hernia due to membranous adhesion of the omentum to the right paracolic gutter.

**Case presentation:**

An 86-year-old female was admitted to our hospital with vomiting and abdominal pain. Laboratory findings showed a slightly elevated C-reactive protein level. Computed tomography scan showed dilated loops of the small intestine in the right paracolic gutter with medial displacement of the cecum and ascending colon. Internal hernia around the cecum due to postoperative adhesion after appendectomy was suspected, and she underwent emergency laparotomy. Intraoperative findings revealed the adhesion between the omentum and right paracolic gutter forming a cavity with the small intestine incarcerated. No abnormal adhesion in the ileocecal region was seen. We transected the omental adhesion from the orifice to the far end of the cavity near the hepatic flexure of the colon to release strangulation and to prevent recurrence. The patient was discharged on postoperative day 14 without complications.

**Conclusions:**

Paracecal hernias have a type of membranous adhesion of the omentum to the right paracolic gutter. Surgeons should be aware of this paracecal hernia type, when they encounter the internal hernia.

## Background

Internal hernias are an infrequent cause of small bowel obstruction [1]. Paracecal hernias, also known as pericecal hernias, are an exceptionally rare type of internal hernia [2]. In a paracecal hernia, herniation generally occurs through an orifice that develops from the peritoneal recess formed by folds of the peritoneum in the paracecal area [3]. We herein report a unique case of paracecal hernia which occurred due to membranous adhesion of the omentum to the right paracolic gutter, along with pertinent literature review.

## Case presentation

**Fig. 1:**
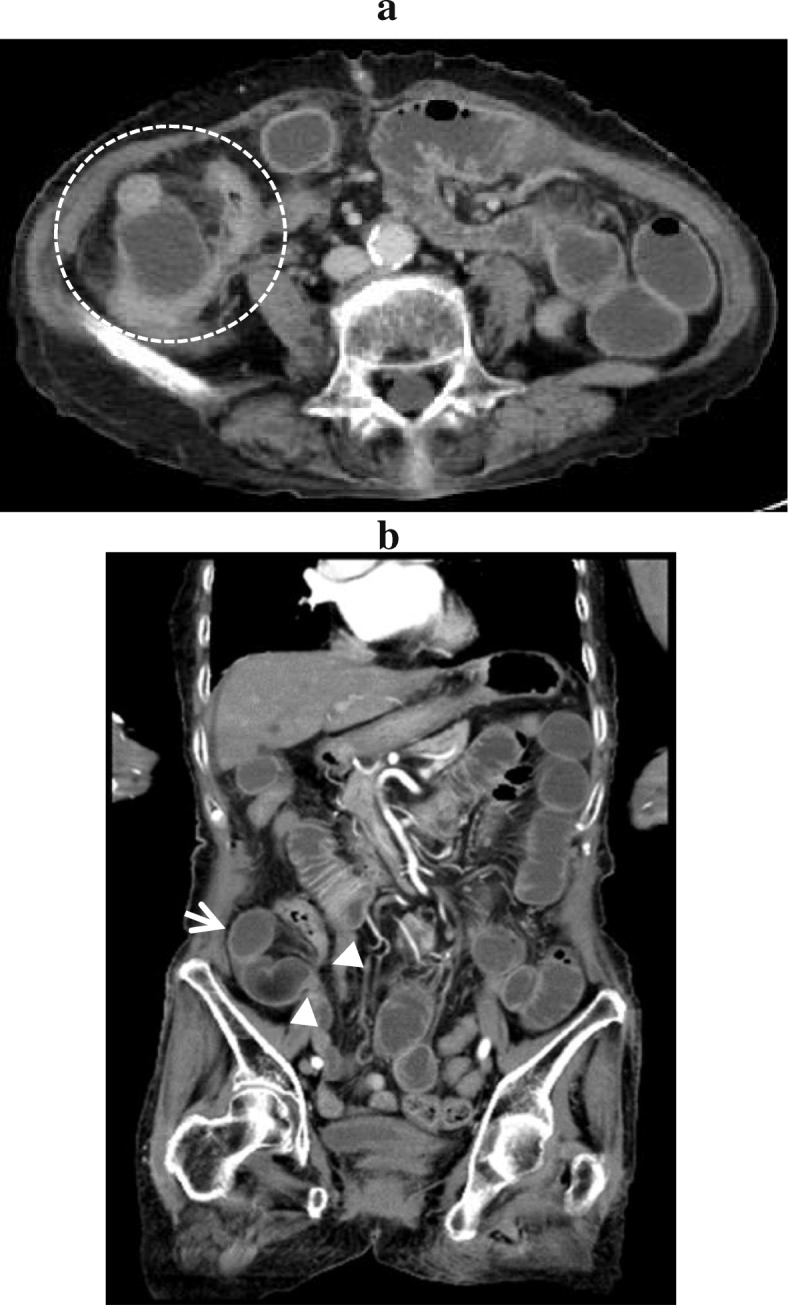
Abdominal enhanced computed tomography (CT) scan on admission. **a** Axial CT scan shows the small intestine in the right paracolic gutter and medially displaced ascending colon. **b** Coronal CT scan shows the closed loop sign of the small intestine in the right paracolic gutter (arrow) and hernia orifice (arrow head)

An 86-year-old female was admitted to our hospital, presenting with vomiting and abdominal pain that had lasted for 2 days. Her past history included McBurney’s appendectomy in her 30s, systemic lupus erythematosus diagnosed in her 50s, and senile dementia for the last 3 years. She had been treated with steroids for a year in her mid-50s but has not received steroids for the last 30 years. She had mild tenderness at the right lower abdomen, without rebound tenderness or guarding. Laboratory findings were only notable for an elevated C-reactive protein level of 0.63 mg/dl. An enhanced computed tomography (CT) scan showed dilated loops of the small intestine in the right paracolic gutter, which displaced the cecum and ascending colon medially (Fig. 1a, b). These findings led us to suspect small bowel obstruction caused by internal hernia around the cecum due to postoperative adhesion after appendectomy. She underwent emergency laparotomy.

**Fig. 2:**
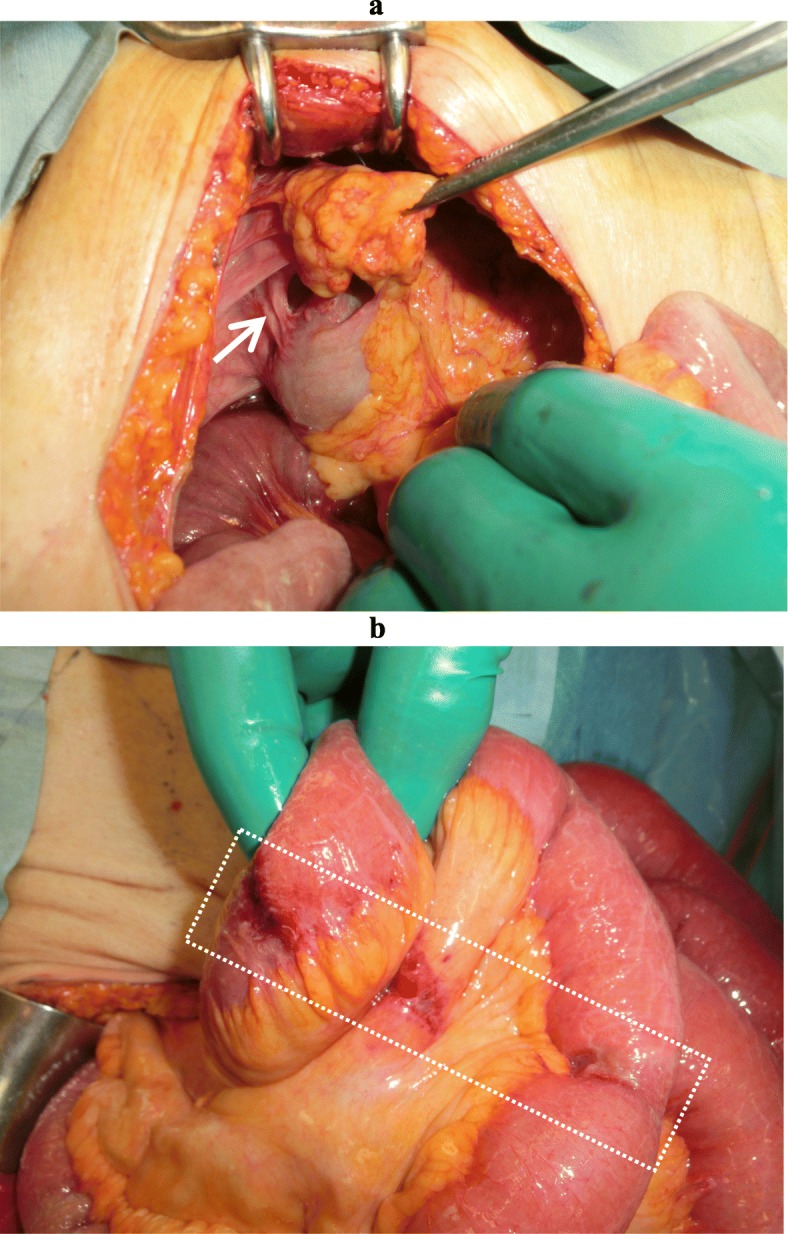
Intraoperative findings. **a** The hernia orifice located lateral to the ascending colon in the right paracolic gutter and posterior to the omentum (arrow). **b** The strangulated part of the closed loop bowel revealed no stenosis or ischemic change

**Fig. 3:**
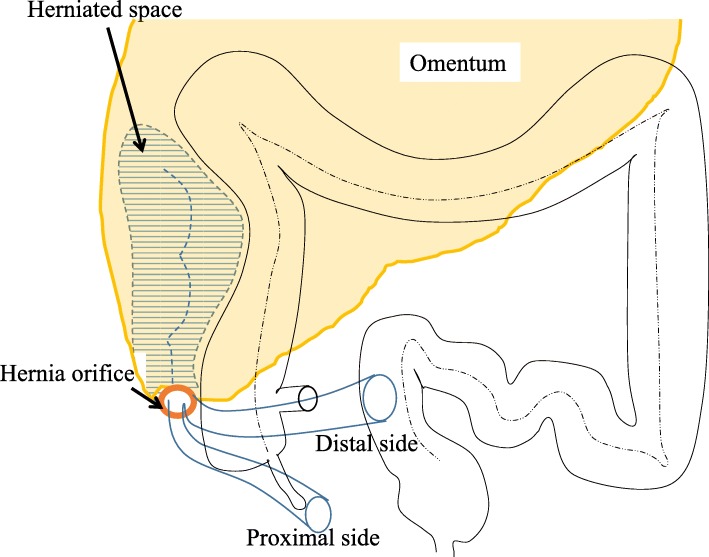
Schematic representation of intraoperative findings

Intraoperative findings revealed the adhesion between the omentum and right paracolic gutter forming a cavity with the small intestine incarcerated (Fig. 2a, b). The cavity was bounded anteriorly by the omentum, posteriorly by the retroperitoneum, laterally by the parietal peritoneum, and medially by the ascending colon (Fig. 3). There was no abnormal adhesion in the ileocecal region, in contrast to this unique adhesion of the omentum along the ascending colon. The omentum was attached from the cecum to the hepatic flexure in a linear manner and naturally transited to the attachment of the transverse colon, suggesting the adhesion was congenital. We opened the orifice to release strangulation and transected omental adhesion from the orifice to the far end of the cavity near the hepatic flexure of the colon to prevent recurrence. The incarcerated small intestine was viable and we did not perform resection of the intestine. The patient was discharged on postoperative day 14 without complications.

## Discussion

Internal hernia is defined as protrusion of abdominal organs into the foramen or recess within the abdominal cavity, and it accounts for 0.5–3% of all cases of intestinal obstruction [4]. Internal hernias are further classified into six types: paraduodenal, foramen of Winslow, paracecal, intersigmoid, transmesenteric or transmesocolic, and retroanastomotic [4]. Paracecal hernias account for 13% of all internal hernias [5].

In a PubMed search of the literature published from January 1980 to August 2019 using the keywords “paracecal hernia,” “retrocecal hernia,” “pericecal hernia,” and “ileocecal hernia,” we found 27 English language reports, describing 33 surgical cases of paracecal hernias including our case (Table 1). The median patient age was 67 years (range 0–92 years) and the male-to-female ratio was 5:6. Five cases had a history of abdominal surgery. Bowel resection was performed in five cases.

**Table 1:** Literature review of paracecal hernia cases from 1980 to 2019

No.	Authors	Reference	Year	Age	Sex	History of abdominal surgery	Meyer’s classification	Orifice	Surgical approach	Resection of bowel	Treatment of the hernia orifice
1	Rosen	[6]	1982	80	F	None	Retrocecal recess type	Retrocecal recess	Laparotomy	–	Closure
2	Rivkind	[3]	1986	0	F	NA	Lateral type	Paracolic sulci	Laparotomy	–	NA
3	Rivkind	[3]	1986	8	M	NA	Lateral type	Paracolic sulci	Laparotomy	–	Open
4	Rivkind	[3]	1986	25	M	NA	Lateral type	Paracolic sulci	Laparotomy	–	NA
5	Rivkind	[3]	1986	77	F	NA	Lateral type	Paracolic sulci	Laparotomy	–	NA
6	Rivkind	[3]	1986	83	F	NA	Lateral type	Paracolic sulci	Laparotomy	–	Closure
7	Lindsey	[7]	1997	86	F	NA	Retrocecal recess type	Retrocecal recess	Laparoscopy	–	Open
8	Patterson	[8]	2000	59	M	None	Lateral type	Paracolic sulci	Laparotomy	–	NA
9	Lu	[9]	2002	69	M	None	NA	NA	Laparotomy	–	NA
10	Lu	[9]	2002	67	F	Appendectomy	NA	NA	Laparotomy	–	NA
11	Omori	[10]	2003	90	F	None	NA	NA	Laparoscopy	–	Closure
12	Osadchy	[1]	2005	76	M	None	Lateral type	Paracolic sulci	Laparotomy	–	Closure
13	Fu	[11]	2006	34	M	None	Internal type	Inferior ileocecal recess	Laparotomy	–	Closure
14	Molto Aguado	[12]	2007	59	F	None	Lateral type	Paracolic sulci	Laparotomy	+	Closure
15	Hirokawa	[13]	2007	74	M	Appendectomy	Retrocecal recess type	Retrocecal recess	Laparoscopy-assisted	–	Open
16	Kabashima	[14]	2010	43	F	Invagination	Lateral type	Paracolic sulci	Laparoscopy	–	Open
17	Shibuya	[15]	2010	63	M	NA	Retrocecal recess type	Retrocecal recess	Laparotomy	–	Closure
18	Choh	[16]	2010	65	F	None	NA	NA	Laparotomy	+	Closure
19	Jang	[17]	2011	84	F	None	Lateral type	Paracolic sulci	Laparotomy	–	Open
20	Nishi	[18]	2011	70	F	None	Lateral type	Paracolic sulci	Laparotomy	–	Open
21	Kleyman	[19]	2013	34	F	None	NA	NA	Laparotomy	–	Closure
22	Saygin	[20]	2015	50	F	None	Lateral type	Paracolic sulci	Laparoscopy	–	NA
23	Kumar	[21]	2015	88	F	None	Internal type	Inferior ileocecal recess	Laparotomy	+	Ileocecal resection
24	Sasaki	[22]	2016	65	M	None	Retrocecal recess type	Retrocecal recess	Laparoscopy	–	Closure
25	Ogami	[23]	2016	92	M	Cholecystectomy	Retrocecal recess type	Retrocecal recess	Laparoscopy	–	Open
26	Ito	[24]	2017	83	M	None	Retrocecal recess type	Retrocecal recess	Laparotomy	–	Open
27	Chia	[25]	2017	32	M	None	Retrocecal recess type	Retrocecal recess	Laparoscopy	–	Open
28	Tayaran	[26]	2017	75	F	None	Lateral type	Paracolic sulci	Laparoscopy	–	Closure
29	Inukai	[27]	2018	54	M	None	Lateral type	Paracolic sulci	Laparoscopy-assisted	+	Open
30	Menezes	[28]	2018	40	M	None	NA	NA	Laparotomy	–	Open
31	Otani	[29]	2018	83	F	None	Lateral type	Paracolic sulci	Laparoscopy	–	Open
32	Aljaberi	[30]	2019	16	M	None	Internal type	Superior ileocolic recess	Laparotomy converted from laparoscopy	+	Ileocecal resection
33	Present case		2019	86	F	Appendectomy	Lateral type	Membranous adhesion of the omentum	Laparotomy	–	Open

*NA* not available, *M* male, *F* female

Meyer et al. [4] have classified paracecal hernia into four subtypes as internal type, retrocecal recess type, lateral type, and unclassifiable type (cecal recess type). In our case, the hernia orifice was located at the right lateral side of the cecum, and a loop of the small intestine was incarcerated in the right paracolic gutter, pushing the ascending colon medially. According to the Meyer’s classification, our case was considered to be a lateral-type paracecal hernia. Previous cases of lateral types have arisen in a peritoneal recess, and the herniation into the space surrounded by the omentum and paracolic gutter like our case has not been reported (Table 1).

Some mechanisms that may cause paracecal hernia have been suggested. One possibility is that the hernial orifice is a congenital anatomic structure. The anatomy of the paracecal peritoneum is attributed to ileocecal migration that occurs during intestinal rotation of the midgut in the fifth month of gestation. Adhesion of the ascending colon and mesentery to the retroperitoneum causes four kinds of peritoneal recesses: superior ileocecal recess, inferior ileocecal recess, retrocecal recess, and paracolic sulci [3, 31]. All of these recesses may become hernial orifices. Another possibility is acquired mechanisms, such as postsurgical or traumatic defects of the mesentery or omentum, postoperative adhesions, and increased pressure in the abdominal cavity related to obesity, coughing, or straining [9, 32]. In the present case, we observed that the small intestine was constrained in the right paracolic gutter covered with the omentum that adhered to the right abdominal wall. The omental attachment was seen from the level of the cecum to the hepatic flexure in a linear manner and naturally transited to the attachment at the transverse colon.

It has been reported that the omental attachment varies among individuals and may extend to the ascending colon [33]. In the development process, the omentum fuses with transverse colon first close to the hepatic flexure of the colon, second in the region of spleen, and last in the middle of the transverse colon until the 14th week of gestation. At 5 years of age, most of the intestines are covered by the omentum, which also extends beyond the flexures of the colon. This extended omentum might contact and be attached to the right abdominal wall in parallel along the ascending colon. Although our case had a history of appendectomy, adhesions were not seen in the ileocecal region in contrast to the reported case in which an adhesion band was seen at the wound of the previous McBurney appendectomy [3]. Thus, we have concluded that the paracecal hernia in our case arose from congenital adhesion of the omentum to the right paracolic gutter.

The clinical symptoms of paracecal hernia are the same as those of small bowel obstruction, namely abdominal pain, nausea, vomiting, constipation, and obstipation [1]. Currently, CT is an important tool for the evaluation of intestinal obstruction and acute abdominal diseases [31] and has become the first-line imaging technique in patients with suspected internal hernia. The CT findings of paracecal hernia include an encapsulated cluster of dilated small bowel loops interposed between the cecum and the abdominal wall, and mesenteric vessels converging toward the entrance of the hernia [1]. CT also gives the information to diagnose the types of paracecal hernia: a lateral shift of the ascending colon in internal type and an anterior shift in retrocecal recess type [34]. In our case, the internally displaced ascending colon was consistent with the lateral type.

Radical therapy for internal hernia is urgent surgery. The reduction of the strangulation is the first step when intestinal ischemia is suspected. Secondly, opening or closure of the hernia orifice is mandatory to prevent recurrence, although it is controversial whether the orifice should be left open or closed (Table 1). We opened the orifice by dissecting the omental attachment because the cavity was too deep to be closed completely without leaving a vacant space. Recently, laparoscopic surgery has been adopted for small bowel obstructions in favor of its high diagnosis rate and minimal invasiveness [27]. We performed open laparotomy for our patient because the dilated bowel was considered to fill up the abdominal cavity, leaving little room to move instruments, increasing the risk of iatrogenic bowel injury [35]. Nevertheless, preoperative diagnosis is indicated for an optimal surgical treatment.

## Conclusion

We describe a unique case of paracecal hernia in which the internal hernia was due to membranous adhesion of the omentum to the right paracolic gutter. Surgeons should be aware of this paracecal hernia type, when they encounter the internal hernia.

## Data Availability

Data sharing is not applicable to this article as no datasets were generated or analyzed during the current study.

## Abbreviation

CT: Computed tomography

## References

[1] Osadchy A, Keidar A, Zissin R (2005). Small bowel obstruction due to a paracecal hernia computerized tomography diagnosis. Emerg Radiol.

[2] Mathieu D, Luciani A (2004). Internal abdominal herniations. AJR Am J Roentgenol.

[3] Rivkind AI, Shiloni E, Muggia-Sullam M, Weiss Y, Lax E, Freund HR (1986). Paracecal hernia: a cause of intestinal obstruction. Dis Colon Rectum.

[4] Meyers MA, Meyers MA (2000). Internal abdominal hernias. Dynamic radiology of the abdomen.

[5] Ghahremani GG (1984). Internal abdominal hernias. Surg Clin North Am.

[6] Rosen L, Woldenberg D, Friedman IH (1981). Small-bowel obstruction secondary to pericecal hernia. Dis Colon Rectum.

[7] Lindsey I, Nottle PD (1997). Laparoscopic management of small bowel obstruction caused by a retrocaecal hernia. Surg Laparosc Endosc.

[8] Patterson R, Klassen G (2000). Small bowel obstruction from internal hernia as a complication of colonoscopy. Can J Gastroenterol.

[9] Lu HC, Wang J, Tsang YM, Tseng HS, Li YW (2002). Pericecal hernia: a report of two cases and survey of the literature. Clin Radiol.

[10] Omori H, Asahi H, Inoue Y, Irinoda T, Saito K (2003). Laparoscopic paracecal hernia repair. J Laparoendosc Adv Surg Tech A.

[11] Fu CY, Chang WC, Lu HE, Su CJ, Tan KH (2007). Pericecal hernia of the inferior ileocecal recess: CT findings. Abdom Imaging.

[12] Molto Aguado M, Gonzalez Valverde FM, Barreras Mateos JA, Vazquez Rojas JL (2007). Small intestinal strangulation due to a primary internal paracecal hernia. Hernia.

[13] Hirokawa T, Hayakawa T, Tanaka M, Okada Y, Sawai H, Takeyama H (2007). Laparoscopic surgery for diagnosis and treatment of bowel obstruction: case report of paracecal hernia. Med Sci Monit.

[14] Kabashima A, Ueda N, Yonemura Y, Mashino K, Fujii K, Ikeda T (2010). Laparoscopic surgery for the diagnosis and treatment of a paracecal hernia repair: report of a case. Surg Today.

[15] Shibuya H, Ishihara S, Akahane T, Shimada R, Horiuchi A, Aoyagi Y (2010). A case of paracecal hernia. Int Surg.

[16] Choh NA, Rasheed M, Jehangir M (2010). The computed tomography diagnosis of paracecal hernia. Hernia.

[17] Jang EJ, Cho SH, Kim DD (2011). A case of small bowel obstruction due to a paracecal hernia. J Korean Soc Coloproctol.

[18] Nishi T, Tanaka Y, Kure T (2011). A case of pericecal hernia with a hernial orifice located on the lateral side of the cecum. Tokai J Exp Clin Med.

[19] Kleyman S, Ashraf S, Daniel S, Ananthan D, Sanni A, Khan F. Pericecal hernia: a rare form of internal hernias. J Surg Case Rep. 2013. 10.1093/jscr/rjs021. 10.1093/jscr/rjs021 PMC5912703 24964406

[20] Saygin H, Kara K, Sari S, Sucullu I, Sonmez G (2015). Education and imaging. Gastrointestinal: a rare cause of small bowel obstruction, paracecal hernia. J Gastroenterol Hepatol.

[21] Kumar S, Dikshit P, Bhaduri S, Sattavan S (2015). Gangrenous appendicitis: a rare presentation of pericecal hernia; case report and review of the literature. Bull Emerg Trauma.

[22] Sasaki K, Kawasaki H, Abe H, Nagai H, Yoshimi F (2016). Retrocecal hernia successfully treated with laparoscopic surgery: a case report and literature review of 15 cases in Japan. Int J Surg Case Rep.

[23] Ogami Takuya, Honjo Hirotaka, Kusanagi Hiroshi (2016). Pericecal hernia manifesting as a small bowel obstruction successfully treated with laparoscopic surgery. Journal of Surgical Case Reports.

[24] Ito S, Takeda R, Kokubo R, Sakai Y, Matsuzawa H, Sugimoto K (2017). Retrocecal hernia preoperatively diagnosed by computed tomography: a case report. Int J Surg Case Rep.

[25] Chia DKA, Tay KV, Kow A, So J, Shabbir A, Kim G (2019). Paracaecal hernia: uncommon but important cause of small bowel obstruction successfully managed with laparoscopic surgery. ANZ J Surg.

[26] Tayaran A, Abdulrasool H, Bui HT (2017). Paracaecal hernia: a case report on the evolving role of laparoscopy. Int J Surg Case Rep.

[27] Inukai K, Tsuji A, Uehara S (2018). Paracecal hernia with intestinal ischemia treated with laparoscopic assisted surgery. Int J Surg Case Rep.

[28] Menezes Richard, Kamble Ranjeet, Joshi Anagha, Chaudhari Kalpesh (2018). Closed loop small bowel obstruction due to paracaecal internal herniation: a lesson in rarity. BMJ Case Reports.

[29] Otani H, Makihara S (2018). Laparoscopic surgery for small bowel obstruction due to paracecal hernia. Acta Med Okayama.

[30] AlJaberi LM, Salameh AK, Mashalah RM, AbuMaria A (2019). Pericecal hernia in a pediatric patient: case report and literature review. Int J Surg Case Rep.

[31] Eun-Jung J, Seung HC, Dae-Dong K (2011). A case of small bowel obstruction due to a paracecal hernia. J Korean Soc Coloproctology.

[32] Lassandro F, Iasiello F, Pizza NL, Valente T, Stefano ML, Grassi R (2011). Abdominal hernias: radiological features. World J Gastrointest Endosc.

[33] Liebermann MD (2000). The greater omentum. Anatomy, embryology, and surgical applications. Surg Clin North Am.

[34] Suyama M, Yasuno M, Takahashi H, Wakayama T (2013). A case report of lateral paracecal hernia (in Japanese). J Jpn Surg Assoc.

[35] Suter M, Zermatten P, Halkic N, Martinet O, Bettschart V (2000). Laparoscopic management of mechanical small bowel obstruction: are there predictors of success or failure?. Surg Endosc.

